# Tracking arctic marine mammal resilience in an era of rapid ecosystem alteration

**DOI:** 10.1371/journal.pbio.2006708

**Published:** 2018-10-09

**Authors:** Sue E. Moore, Randall R. Reeves

**Affiliations:** 1 National Oceanic and Atmospheric Administration Fisheries, Office of Science and Technology, Seattle, Washington, United States of America; 2 Okapi Wildlife Associates, Hudson, Quebec, Canada

## Abstract

Global warming is significantly altering arctic marine ecosystems. Specifically, the precipitous loss of sea ice is creating a dichotomy between ice-dependent polar bears and pinnipeds that are losing habitat and some cetaceans that are gaining habitat. While final outcomes are hard to predict for the many and varied marine mammal populations that rely on arctic habitats, we suggest a simplified framework to assess status, based upon ranking a population’s size, range, behavior, and health. This basic approach is proposed as a means to prioritize and expedite conservation and management efforts in an era of rapid ecosystem alteration.

This Perspective is part of the *Confronting Climate Change in the Age of Denial Collection*.

## Introduction

Arctic marine ecosystems are changing fast, as manifested by loss of sea ice, ocean warming and freshening, storm prevalence and severity, and regional increases in primary productivity [1, 2]. The dramatic decline of sea ice thickness, extent, and duration is a key indicator of rapid ecosystem alteration. Images of starving polar bears (*Ursus maritimus*) or thousands of walruses (*Odobenus rosmarus*) massed together on beaches instead of hauled out on ice floes signal hard times for these species. Indeed, the polar bear has become a “poster species” for global warming, used by organizations either to (i) attract attention and funds by linking the bear’s plight to that of nature as a whole or (ii) project images of healthy bears in an effort to discredit the irrefutable evidence of rapid planetary warming [3]. Arctic marine mammals rely on sea ice in a variety of ways, depending on their life history and behavioral ecology [4]. Polar bears use ice as a platform for resting, walking, and stalking seals; walruses and ice seals for pupping, nursing, molting, and resting. Sea ice loss means less suitable habitats for them. In contrast, increased primary and secondary productivity associated with the reduction in sea ice opens new feeding opportunities for cetaceans, including both endemic arctic species and species that migrate seasonally to arctic waters [5]. We recognize that this dichotomous portrayal is simplistic. For example, arctic cetaceans also benefit from sea ice, as it can protect them from predators [6] and likely reduces competition with seasonally migrant species [5]. Further, increased productivity would be expected to improve foraging for at least some pinnipeds and polar bears; however, on the whole, species requiring sea ice as a platform are the most challenged by its loss.

Marine mammals are ecosystem sentinels, capable of reflecting ocean variability through changes in their ecology and body condition [4]. Eleven species are endemic to the Arctic—3 cetaceans, 7 pinnipeds, and the polar bear [7]. At least 5 cetacean species migrate to arctic waters, principally to feed in summer and autumn months. Combined, these endemic and seasonally migrant species exhibit a wide range of life history traits that provide a varied phenotypic landscape for natural selection in the Arctic’s regionally diverse and strongly seasonal habitats [8]. In such a setting, an overarching question is what capacity do arctic endemic species have to adapt to ecosystem alterations caused by rapid warming? Specifically, what aspects of their life histories contribute to resilience and can their status as ecosystem sentinels be harnessed to inform and guide conservation efforts?

Here, we propose a basic framework to both broaden and simplify metrics used to assess marine mammal population status as a means to prioritize and expedite urgently needed conservation and management actions in a rapidly changing Arctic. We briefly summarize evidence from decades-long studies of a few marine mammal species and identify features common to populations that appear to be doing well, and those that are not, in the face of rapid habitat alteration due to climate change. We use those features to broaden the discussion to matters related to species resilience, including the importance of (i) population size, (ii) seasonal range, (iii) behavioral plasticity, and (iv) health. We then propose a simplified approach to assess population status based on summed rankings of those 4 resilience metrics. The overarching goal is to use the resultant scores to prioritize management and conservation actions. While we do not delve here into specific anthropogenic threats to arctic marine mammals, such as those associated with offshore commercial activities [9, 10, 11], we recognize these activities as important contributors to ecosystem alteration. Similarly, while recognizing that complexity-focused approaches to marine mammal research and conservation are poised to advance [12], we suggest a simplified approach that incorporates multiple facets of animal ecology and health as an achievable step in the near future. We close with thoughts on the recent recognition of marine mammals as “essential ocean variables” in a program to monitor biodiversity and ecosystem changes through sustained ocean observation [13].

### Winners and losers

The bowhead whale (*Balaena mysticetus*) is the only baleen whale endemic to the Arctic. Bowheads are long lived (to approximately 200 years [14]) and fully adapted to arctic conditions, e.g., capable of breaking through sea ice (up to 18 cm thick) to breathe. Thus, it may seem counterintuitive that this pagophilic (ice-loving) species appears to be thriving during a period of rapid sea ice loss, at least in the Pacific Arctic region. There, population size has grown, calf counts have increased, and body condition of individual whales has improved over the last quarter-century [15, 16]. These positive outcomes have been attributed to overall expansion of primary production and an augmented food supply for bowheads due to increased zooplankton advection into the Pacific Arctic, accompanied by upwelling of prey during the extended open-water season [5]. While still recovering from over-harvest during the commercial whaling era, bowheads in the Davis Strait–Baffin Bay region appear to be increasing [7], and copious singing (up to 24 h/day) recorded throughout the winter in Fram Strait east of Greenland suggests a rebounding population there [17].

The situation for the beluga, or white whale (*Delphinapterus leucas*), is less clear. Trends in abundance are known for only 6 of 22 populations [18], and studies of diet and body condition are rare [19, 20, 21]. Beluga populations can be either local or migratory [18], with those that are local often considered to be at greater risk. Of note, habitat selection by 2 populations that undertake long migrations in the Pacific Arctic was associated primarily with bathymetric features rather than ice conditions during the recent period of sea ice loss [22]. A trend in one population toward longer, deeper dives was thought to be an indirect effect of sea ice loss, assuming that ecological changes shifted foraging opportunities to deeper water.

Cetaceans that migrate seasonally to arctic waters are also considered winners. In the Pacific Arctic, the gray whale’s (*Eschrichtius robustus*) use of continental shelf habitats has been the focus of study since the 1980s [23], during which time population size has steadily increased [24]. With the open-water season extended by 2–4 weeks [6], humpback (*Megaptera novaeangliae*), fin (*Balaenoptera physalus*), and minke whales (*B*. *acutorostrata*) are now commonly seen north of Bering Strait, unlike 30 years ago [25]. The recent surge in sightings is probably due to a combination of increased survey effort, the growth of whale populations previously hunted commercially, and climate-driven environmental changes. These same 3 baleen whale species have long been common in parts of the Atlantic Arctic [26], especially the Barents Sea [27]. Competition for prey between them and the endemic bowhead may be mediated, at least in the near term, by differences in migration timing, prey preferences, and feeding behavior [5].

Polar bears are the iconic “losers” in reports of climate change impacts [3], with walruses and ringed seals (*Pusa hispida*) running close behind. While some polar bear populations show signs of stress, including extreme emaciation and reproductive failure, others still appear to be in good body condition [28]. Continued loss of sea ice will lead to range contraction and increasing isolation of some populations [29], and it is clear that polar bears rely on a fat-rich diet of marine mammals that cannot be easily obtained on land [30]. Walruses, ringed seals, and bearded seals (*Erignathus barbatus*) are also rapidly losing sea ice habitat, but while these pagophilic species may have to swim farther to feed, they have some capacity to adapt by hauling out on land, as walruses in the Atlantic Arctic and Russia often do [31] and as ringed and bearded seals sometimes do in Svalbard [32], parts of the Okhotsk Sea [33], and Alaska [34]. The endemic narwhal (*Monodon monoceros*) is another loser in the rapidly changing environments of the eastern Canadian Arctic, Greenland, and Svalbard [35]. With their restricted distribution and strong fidelity to pack-ice habitat where they feed in winter, narwhals are considered the most specialized of arctic cetaceans [35]. The extreme physiological adaptations of their skeletal muscles may make them especially sensitive to climate change [36].

### Resilience

**Fig 1:**
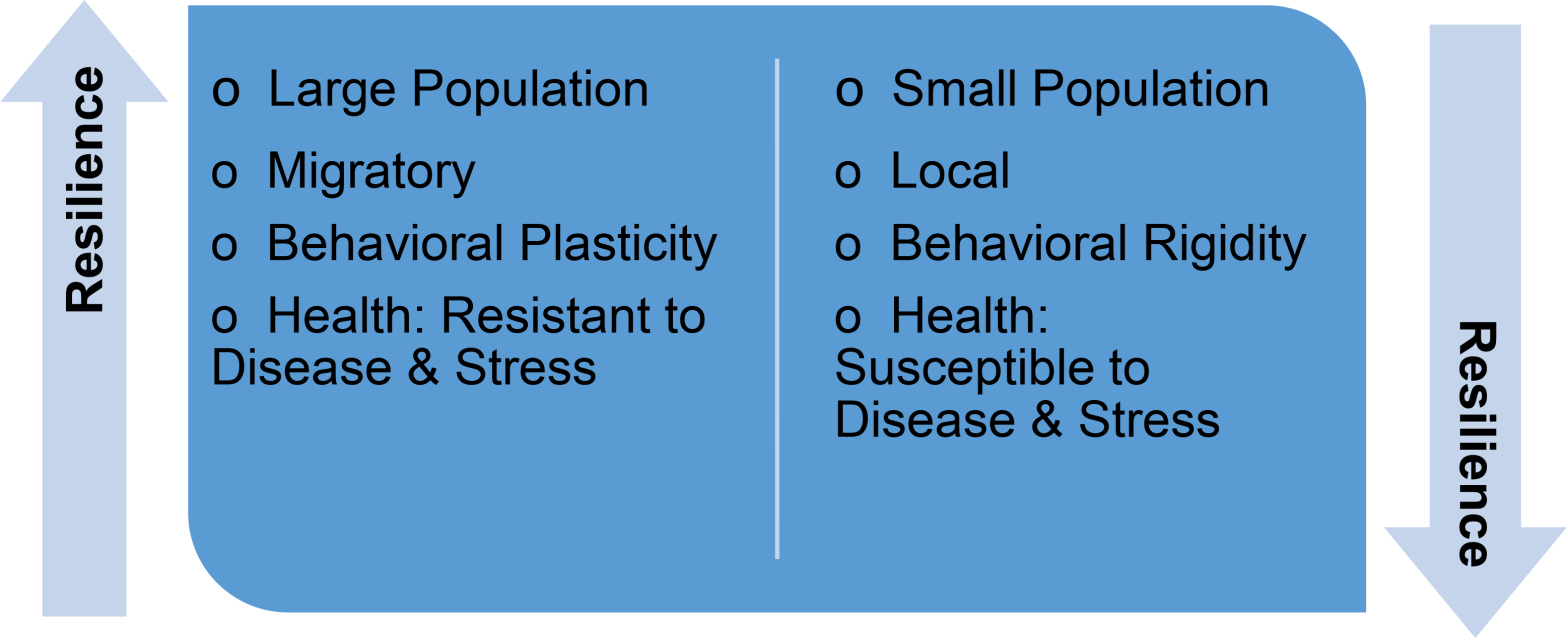
The resilience of marine mammal populations can be assessed based on population size, range, behavioral plasticity, and health. Each of these 4 metrics can be ranked from 1 to 5, with a rank of 1 representing large, migratory populations exhibiting behavioral plasticity (including diet) and resistance to disease and stress and a rank of 5 representing the opposite.

Resilience, in the present context, denotes the capacity to adapt to environmental change. Fundamentally, a population’s resilience to habitat alteration depends on a combination of how it responds to perturbation (adapts, moves) and how sensitive it is to perturbation (life history traits, physiological limits) [37]. Generalist foragers with broad distributions are usually considered more resilient than feeding specialists with a restricted range [38]. A simple index of resilience can be devised based upon 4 metrics: (i) population size, (ii) range, (iii) behavior, and (iv) health (Fig 1). Greater resilience is associated with large populations that display behavioral flexibility (including diet) and show resistance to disease and stress, while the reverse generally signifies lesser resilience. We suggest applying a simple 5-point ranking scale, whereby a score of 1 denotes a large, wide-ranging population that displays considerable behavioral plasticity and resistance to disease, while a score of 5 indicates the opposite. By this method, the bowhead whale population in the Pacific Arctic would receive a score of 1, while the smaller Eastern Canada–West Greenland and Svalbard–Barents Sea populations might score as 3 and 4, respectively. Similarly, large, wide-ranging polar bear and beluga populations would have a lower score (i.e., higher resilience) than small, local populations (i.e., lower resilience). This method of ranking resilience is similar to the sensitivity index developed a decade ago [34], but it is based on only 4 metrics (instead of 8), one of which is related to animal health [39]. While we recognize that such simplification brings the risk of overlooking or obscuring factors that could prove decisive in a given instance (e.g., loss of critical habitat or phenotypic uniqueness), the ranking method’s strength lies in the capacity of marine mammals to integrate and reflect complex ecosystem changes through their ecological and physiological responses [4].

### Tracking arctic marine mammal resilience

**Fig 2:**
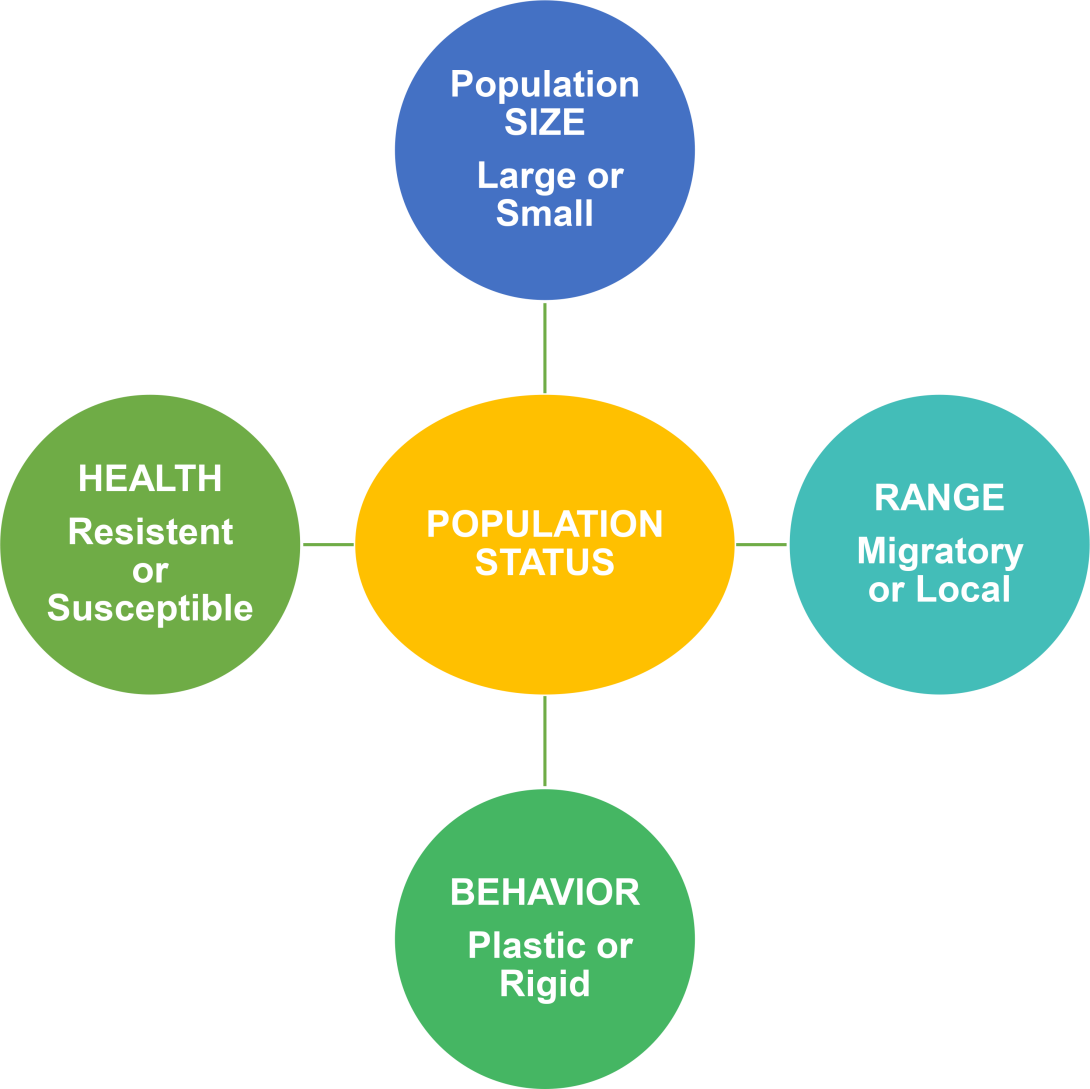
Contribution of 4 metrics to an assessment of a marine mammal population’s status; a simplified approach relevant everywhere but needed with special urgency to prioritize and expedite management and conservation actions in the context of ongoing rapid ecosystem alteration in the Arctic.

Arctic marine mammal populations are often difficult to define and count, with comparatively few reliable estimates of numbers or trends [7]. Furthermore, detecting even precipitous declines in marine mammal population size is unlikely because surveys are too infrequent, and the estimates obtained are too imprecise [40]. Given these challenges and the rapid pace of environmental change in the Arctic, a more holistic approach to assess population status is urgently needed to guide conservation and management actions. We suggest a framework that links the best information available on population size (even if imprecise or only qualitative) to the other 3 metrics that contribute to resilience (Fig 2). A key strength of this framework is the inclusion of both ecological (geographical range and behavior) and physiological (health) metrics, which broaden the foundation of population assessment beyond demography alone. Further, the framework relies upon multidisciplinary science, which increases the likelihood of detecting changes in population status. For example, a shift in migratory timing, a switch in diet, or an outbreak of disease could alert resource managers to a problem that would go unnoticed when relying solely on trends in population size [40]. Importantly, the reliance of indigenous people upon arctic marine mammals makes urgent comprehensive marine mammal health monitoring due to food safety concerns [39]. This “marine mammal connection” also creates opportunities to improve understanding of the nature and trajectories of fast-changing ocean ecosystems through partnerships between conventional science practitioners and the holders of indigenous knowledge [41]. Marine mammals were recently recognized as “essential ocean variables” within a Global Ocean Observing System (GOOS) that is relevant for science and public awareness [13]. An arctic marine mammal tracking framework such as we describe could provide the GOOS with essential evidence on the status of these ecosystem sentinels, and the observatory could in turn provide an online portal for quick delivery of information to resource managers. While the goals set forth by GOOS are challenging [13], they offer a path toward sustainability through improved prediction, more precaution, and wiser policy in this era of global environmental change [42].

## Abbreviation

GOOS: Global Ocean Observing System
